# A risk stratification model for coronary artery lesions in Kawasaki disease: focus on subgroup-specific utility

**DOI:** 10.3389/fcvm.2025.1543767

**Published:** 2025-04-29

**Authors:** Chuxiong Gong, Zhongjian Su, Qinhong Li, Hongyan Li, Ziyu Wang, Huiing Gao, Yamin Li, Xiaomei Liu, Lili Deng

**Affiliations:** ^1^ Department of Cardiovascular Medicine, Kunming Children’s Hospital, Kunming, Yunnan, China; ^2^ Department of Cardiology, First Affiliated Hospital of Kunming Medical University, Kunming, Yunnan, China

**Keywords:** Kawasaki disease, nomogram, coronary artery lesions, cardiovascular diseases, children's diseases

## Abstract

**Objective:**

Kawasaki disease is an acute immune vasculitis that often has a poor prognosis when complicated by coronary artery lesions. Our study aims to construct a risk model for Kawasaki disease complicated by coronary artery lesions and validate it in different clinical characteristic subgroups, optimizing personalized and precise management of Kawasaki disease to improve patient outcomes.

**Methods:**

First, we compared each factor between the groups with and without coronary artery damage. We then used LASSO analysis to further filter for factors that were more significant in predicting outcomes. The selected factors were used to construct the risk model. The model was evaluated using ROC curves, calibration curves, and DCA, and was internally validated using 5-fold cross-validation. Finally, we also conducted subgroup analyses based on factors such as age stages and sex.

**Results:**

Through univariate analysis, LASSO analysis, and correlation analysis, we identified WBC, PLT, CRP, ALB, Na, Time to IVIG treatment, and symptoms of limb as the key factors for constructing the risk model. The model achieved an area under the curve of 0.815(95%CI: 0.779–0.851). Additionally, calibration curves, DCA, and 10-fold cross-validation demonstrated that the model has good predictive performance. The predictive efficacy of the model was also satisfactory across various subgroups.

**Conclusions:**

Our study has constructed a risk model for Kawasaki disease complicated by coronary artery lesions in the Chinese population that demonstrates good predictive performance, and it has been validated successfully across multiple subgroups.

## 1 Introduction

Kawasaki disease (KD) is an acute immune-mediated vasculitis that primarily affects children under the age of five. KD is most prevalent in East Asian countries such as China and Japan. Approximately 25% of KD patients experience the most severe complication, coronary artery lesions (CAL) (1). CAL refers to the inflammation and fibrosis of the coronary artery intima caused by Kawasaki disease, leading to arterial stenosis, thrombosis, and subsequent myocardial ischemia (2). CAL is the leading cause of mortality in KD cases. Currently, the diagnosis of KD relies mainly on clinical symptoms such as the duration of fever, mucosal changes, and limb edam, which can lead to delayed or missed diagnoses (3, 4). This is particularly concerning in atypical KD cases, where delays in acute-phase treatment may increase the risk of CAL. Early prediction of whether children with Kawasaki disease will develop coronary artery lesions is crucial, as it can facilitate timely interventions and treatment to mitigate or prevent further coronary artery damage.

However, the pathogenesis of KD remained incompletely understood, making the prediction of its complication, CAL, a challenging area. Studies demonstrated that younger children were more prone to developing CAL (5, 6). Infants and toddlers, in particular, faced a higher risk due to their smaller coronary arteries, which were more susceptible to inflammation. Additionally, research identified certain clinical features and laboratory indicators in the early stages of the disease as associated with the risk of coronary artery lesions in KD patients. These included prolonged high fever, elevated white blood cell counts, increased platelet counts, and significantly elevated CRP levels (7–10).

As a systemic inflammatory condition, KD presents significant clinical variability and prognostic heterogeneity due to diverse patient characteristics. Factors such as gender, age, ethnicity, and disease duration can substantially influence disease progression and treatment response (11). Studies utilizing PCA analysis have identified multiple subgroups within KD, revealing significant differences in CAL outcomes among these subgroups. Therefore, our study aimed to develop a risk model for CAL in KD and validate it across different clinical characteristic subgroups. The reproducibility and universality of these subgroups are still needed. This approach aided not only in identifying clinical features and predictive efficacy for specific patient populations but also provided important insights for the development of personalized treatment strategies. Gaining a deeper understanding of the differences among these subgroups may have optimized the management of Kawasaki disease and improved patient prognosis.

## 2 Materials and methods

### 2.1 Participants

Our study retrospectively collected clinical data from 1,795 cases of Kawasaki disease diagnosed at Kunming Children's Hospital between December 2014 and December 2023. These cases represent an expansion based on previous research. All cases met the criteria for Kawasaki disease established by the American Heart Association (AHA). The study was conducted in accordance with the principles of the Declaration of Helsinki and received approval from the Ethics Committee of Kunming Children's Hospital (Ethics Approval No.: 2023-05-016-K01).

### 2.2 Diagnostic and inclusion/exclusion criteria

Diagnostic Criteria: All cases in our study conformed to the AHA standards for Kawasaki disease, which include complete Kawasaki disease (CKD) and incomplete Kawasaki disease (IKD). Coronary artery lesions (CAL) were defined as a maximum Z-score of the coronary arteries greater than 2.5 within 60 days of onset (12). IVIG non-response was defined as persistent fever above 38°C after 36 h post-standard initial treatment, or recurrent fever within two weeks after initial treatment accompanied by at least one major clinical manifestation of Kawasaki disease, after excluding other potential causes of fever.

Inclusion Criteria: (1) Hospitalized children diagnosed with Kawasaki disease according to the “Expert Consensus on the Diagnosis and Acute Treatment of Kawasaki Disease”; (2) Complete clinical data available for KD patients. Exclusion Criteria: (1) Patients with underlying conditions such as cardiovascular diseases, liver diseases, kidney diseases, hematologic disorders, immune system diseases, and endocrine or metabolic genetic disorders; (2) Children with a history of previous Kawasaki disease; (3) KD patients who received IVIG, aspirin, or glucocorticoids prior to hospitalization; (4) KD patients with incomplete clinical data; (5) CAL occurred before IVIG treatment.

### 2.3 Inclusion factors

The data for our study originated from the results of examinations conducted upon initial hospitalization before any treatment was implemented. The included variables comprised three demographic characteristics (age at onset, gender, and ethnicity), ten laboratory results (hemoglobin (HB), white blood cell count (WBC), platelet (PLT), percentage of neutrophils (N), percentage of lymphocytes (L), erythrocyte sedimentation rate (ESR), C-reactive protein (CRP), alanine aminotransferase (ALT), total bilirubin (TBIL), gamma-glutamyl transferase (GGT), Albumin (ALB), Globulin (GLB), Bile Acid, Potassium (K), Sodium (Na), Chloride (Cl), Magnesium (Mg), Phosphorus (P), Calcium (Ca), Prothrombin Time (PT), Activated Partial Thromboplastin Time (APTT), Fibrinogen (Fbg), Thrombin Time (TT), D-dimer (D.dimer), Immunoglobulin A (IgA), Immunoglobulin M (IgM), and Immunoglobulin G (IgG)), and five physical examination findings (oral mucosal involvement, conjunctival injection, cervical lymphadenopathy, symptoms of limb, and rash). Additionally, data on the number of days with fever (Fever days), days of intravenous immunoglobulin (IVIG) use (Time to IVIG treatment), and echocardiography were included.

### 2.4 Model construction

Initially, all cases were divided into two groups: CAL and nCAL, based on the occurrence of CAL. Univariate statistical analyses were performed on clinical data between the two groups, and variables with *P* < 0.05 were included in subsequent analyses. Next, we used the “glmnet” R package to perform Least Absolute Shrinkage and Selection Operator (LASSO) to further filter the factors that are more important for predicting the outcome. LASSO is a regularization method for linear regression. It achieves variable selection and model simplification by introducing an L1 regularization term into the loss function. The main advantages of LASSO include its ability to shrink the coefficients of unimportant variables to zero, thereby retaining only the key variables, reducing model complexity and preventing overfitting. During the selection process, 5-fold cross-validation was used to select the optimal lambda value, namely lambda.min or lambda.1se. A larger lambda indicates a stronger regularization effect of the model, resulting in fewer selected independent variables. Variables were selected based on the optimal and maximum lambda values. Additionally, we performed correlation analysis on the selected factors using the “corrplot” R package to avoid multicollinearity among highly correlated factors. Subsequently, we utilized the “rmda” R package to conduct multifactorial logistic regression analysis, retaining the optimal model factors as our final key factors. A nomogram of the optimal model was created using the “rms” R package.

### 2.5 Model evaluation and validation

We initially evaluated the model using the “pROC” R package to plot the receiver operating characteristic (ROC) curve, with the area under the curve (AUC) representing the effectiveness of the model. Calibration of the model was assessed using calibration curves generated by the “ResourceSelection” R package, and decision curve analysis (DCA) was performed using the “decision_curve” function to further evaluate the model's accuracy. Finally, we conducted 5-fold cross-validation using the createFolds function.

### 2.6 Subgroup analysis

To evaluate the predictive performance of the model across different subgroups of Kawasaki disease, we categorized the 1,795 KD patients into multiple subgroups. These included sex (male and female), age stages (Infancy, Toddlerhood, Preschool age, Reach 7 years), ethnicity (Ethnic minorities, Han ethnicity), diagnostic criteria based on typical features (IKD and CKD), duration of fever (less than 5 days and 5 days or more), and illness duration in relation to the common increase in platelet count (illness duration less than 1 week and reaching 1 week). We calculated the AUC for each subgroup to assess the model's predictive value.

### 2.7 Statistical analysis

All statistical analyses were performed using R version 4.4.1. For normally distributed continuous data, means were expressed as mean ± sd and independent samples *t*-tests were used for comparisons between groups. Non-normally distributed data were described as median (P_25_, P_75_), and comparisons were conducted using non-parametric rank-sum tests. Categorical data were expressed as rates, with comparisons between groups performed using chi-square tests or Fisher's exact probability method. A *p* < 0.05 was considered statistically significant.

## 3 Results

### 3.1 Clinical characteristics description and comparison of two groups

Our study included a total of 2,686 KD patients. Among them, 12 cases met exclusion criterion (1); 27 cases met exclusion criterion (2); 589 cases met exclusion criterion (3); 433 cases met exclusion criterion (4); and 116 cases met exclusion criterion (5). In the end, a total of 1,795 Kawasaki disease patients who met the diagnostic and inclusion/exclusion criteria with complete clinical data were included. Among the included patients, 1,130 (62.95%) were male and 665 (37.05%) were female. The majority of patients were of Han ethnicity, with 1,524 cases (84.90%), while 271 cases (15.10%) were from ethnic minorities. The average age of the patients was 2.62 years. Based on whether the Z-score was less than 2.5, patients were divided into the CAL group (195 cases, 10.86%) and the nCAL group (1,600 cases, 89.14%).

A comparison of clinical data between the CAL and nCAL groups revealed significant statistical differences in various parameters, including WBC, PLT, ESR, CRP, ALB, Na, fever days, Time to IVIG treatment, HB, PLR, ALT, GGT, bile acid, Ca, IgA, IgM, and symptoms of limb (Table 1). Notably, the type of KD was not included in subsequent analyses as a clinical characteristic.

**Table 1:** Comparison of clinical data between the CAL and nCAL groups.

Factor	Total (*n* = 1,795)	CAL (*n* = 195)	nCAL (*n* = 1,600)	*p*
Age	2.62 ± 2.01	2.88 ± 2.39	2.59 ± 1.95	0.11
WBC	14.19 ± 5.37	18.39 ± 9.03	13.68 ± 4.47	<0.001
PLT	359.24 ± 127.04	474.49 ± 180.88	345.2 ± 111	<0.001
N	61.49 ± 17.38	63.19 ± 16.95	61.28 ± 17.43	0.14
L	28.81 ± 14.82	27.32 ± 14.66	28.99 ± 14.84	0.136
ESR	60.92 ± 28	66.1 ± 28.82	60.29 ± 27.84	0.008
CRP	77.6 ± 50.42	107.08 ± 57.98	74.01 ± 48.22	<0.001
ALB	34.89 ± 3.74	32.64 ± 4.88	35.16 ± 3.47	<0.001
GLB	26.25 ± 5.99	26.02 ± 5.27	26.28 ± 6.08	0.52
Na	135.38 ± 3.23	132.74 ± 4.22	135.7 ± 2.93	<0.001
Cl	99.5 ± 4	99.54 ± 3.94	99.49 ± 4.01	0.857
Mg	0.89 ± 0.15	0.88 ± 0.13	0.89 ± 0.15	0.623
PT	12.8 ± 3.19	12.7 ± 3.3	12.81 ± 3.18	0.651
APTT	32.64 ± 7.19	32.58 ± 7.25	32.65 ± 7.19	0.901
Fbg	5.28 ± 1.42	5.3 ± 1.31	5.27 ± 1.44	0.759
TT	16.64 ± 2.75	16.35 ± 2.41	16.68 ± 2.79	0.081
D-dimer	0.51 ± 6.25	0.36 ± 1.23	0.52 ± 6.61	0.381
Fever days	5.65 ± 2.13	6.6 ± 2.71	5.53 ± 2.01	<0.001
Time to IVIG treatment	6.17 ± 1.85	7.08 ± 2.5	6.06 ± 1.72	<0.001
HB	113 (105–121)	110 (101–118.5)	113 (106–121)	<0.001
NLR	2.38 (1.35–4.16)	2.8 (1.46–4.6)	2.34 (1.34–4.11)	0.104
PLR	101.83 (69.65–147.19)	118.11 (82.65–170.86)	99.9 (68.46–144.58)	<0.001
ALT	28.7 (15–67.45)	43 (20.5–92.5)	27 (14–64)	<0.001
GGT	37 (16–94)	57 (26–129)	34 (15–90)	<0.001
TBIL	9.2 (6.5–17.4)	9.2 (6.2–30.35)	9.2 (6.5–16.5)	0.526
Bile Acid	7.3 (4.8–11.4)	8.2 (5.3–12.8)	7.1 (4.8–11.3)	0.037
K	4.3 (3.8–4.8)	4.2 (3.8–4.8)	4.3 (3.8–4.8)	0.386
P	1.39 (1.17–1.58)	1.38 (1.1–1.55)	1.39 (1.17–1.58)	0.121
Ca	2.23 (2.13–2.33)	2.21 (2.1–2.3)	2.23 (2.13–2.33)	0.007
IgA	0.7 (0.4–0.96)	0.77 (0.46–1.05)	0.69 (0.4–0.95)	0.048
IgM	1.2 (0.84–1.42)	1.2 (0.98–1.45)	1.18 (0.82–1.42)	0.043
IgG	6.9 (4.9–9.18)	7.3 (5.36–9.18)	6.81 (4.88–9.18)	0.061
Sex				0.224
Female	665	64 (32.82)	601 (37.56)	
Male	1,130	131 (67.18)	999 (62.44)	
Ethic				0.39
No	1,524	161 (82.56)	1,363 (85.19)	
Yes	271	34 (17.44)	237 (14.81)	
Oral mucosal involvement				0.999
No	326	35 (17.95)	291 (18.19)	
Yes	1,469	160 (82.05)	1,309 (81.81)	
Conjunctival injection				0.463
No	247	23 (11.79)	224 (14)	
Yes	1,548	172 (88.21)	1,376 (86)	
Rash				0.491
No	501	59 (30.26)	442 (27.62)	
Yes	1,294	136 (69.74)	1,158 (72.38)	
Cervical lymphadenopathy				0.107
No	846	103 (52.82)	743 (46.44)	
Yes	949	92 (47.18)	857 (53.56)	
Symptoms of limb				<0.001
No	936	73(37.44)	863(53.94)	
Yes	859	122(62.56)	737(46.06)	

### 3.2 Screening for key factors

**Figure 1:**
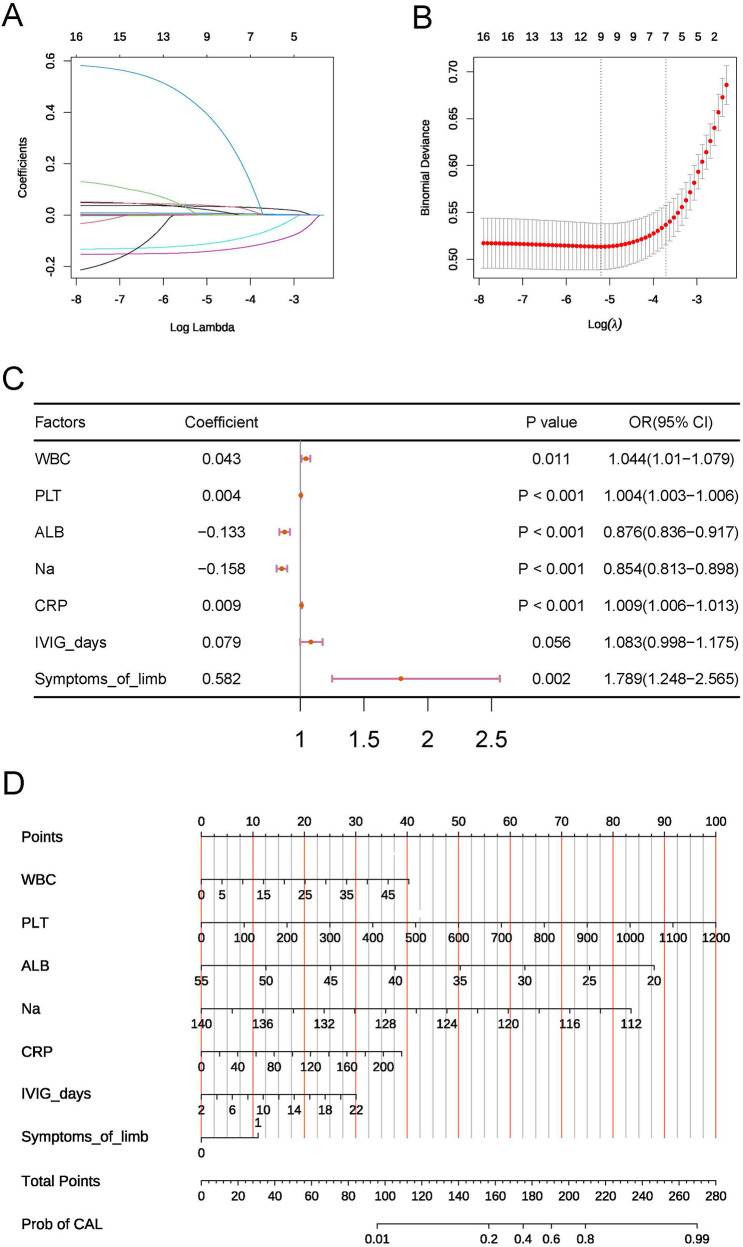
**(A,B)** Key variables selected by LASSO. **(C)** Forest plot of the multivariable logistic regression model. **(D)** Nomogram of the risk model.

To ensure the operational simplicity of the risk model, the aforementioned 13 factors were included in a LASSO analysis to identify those with greater predictive value. In the LASSO analysis, we selected the optimal *λ* value of 0.0244 through 10-fold cross-validation. A larger *λ* value indicates a higher degree of regularization for the model, leading to a reduced number of selected independent variables. Ultimately, we filtered out WBC, PLT, CRP, ALB, Na, Time to IVIG treatment, and symptoms of limb (Figures 1A,B). Therefore, these 7 factors were included in the subsequent model construction.

To address potential multicollinearity, correlation analysis was performed on the seven key factors. Pearson correlation coefficients among all factors were below 0.5 (maximum absolute value = 0.34), indicating a low risk of multicollinearity bias.

### 3.3 Construction and evaluation of the risk model

The seven key factors were incorporated into a multifactorial logistic regression analysis model, and higher WBC, higher PLT, higher CRP, lower ALB, lower serum Na ion levels, longer Time to IVIG treatment, and the presence of symptoms of limb were identified as independent risk factors for KD complicated by CAL (Figure 1C). We also constructed a nomogram to visualize the risk model (Figure 1D).

**Figure 2:**
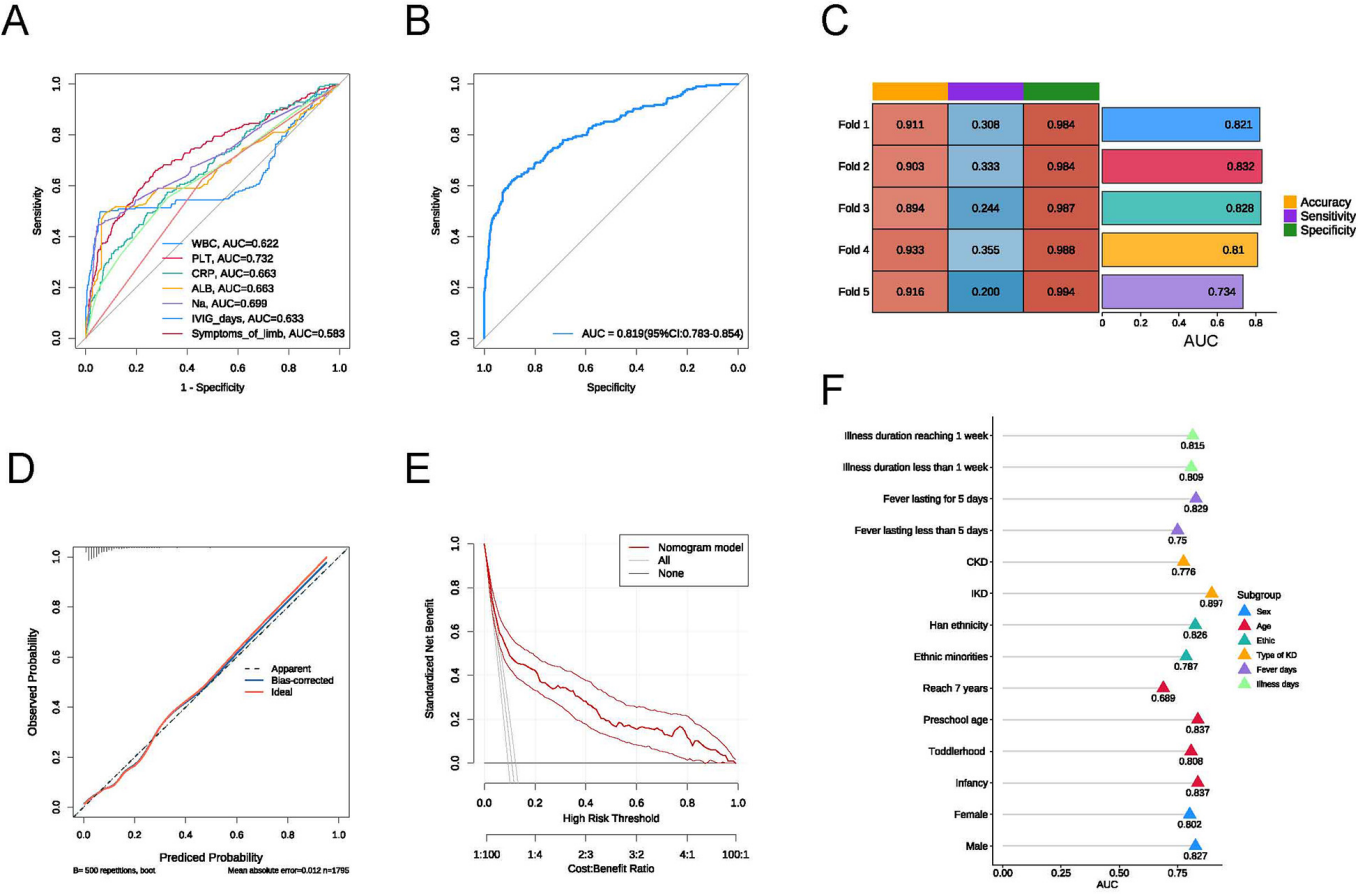
**(A)** ROC curve of the risk model; **(B)** ROC curves for individual factors: WBC, PLT, CRP, ALB, Na, time to IVIG treatment, and symptoms of limb; **(C)** results of the 5-fold cross-validation; **(D)** calibration curve of the risk model; **(E)** DCA curve of the risk model; **(F)** AUC values for each subgroup.

To assess the predictive efficacy of the model, we first plotted the ROC curves for the seven independent risk factors, with PLT showing the highest AUC value of 0.732 (Figure 2A). We then plotted the ROC curve for the multifactorial logistic regression model, which yielded an AUC value of 0.819 (95% CI: 0.783–0.854), an accuracy of 0.911, a sensitivity of 0.282, and a specificity of 0.988 (Figure 2B). The calibration curve demonstrated satisfactory consistency for the CAL risk prediction model (Figure 2C), and the DCA further confirmed the model's accuracy (Figure 2D). To validate the predictive performance of the model, we conducted 5-fold cross-validation, which indicated that the constructed model has good predictive efficacy (Figure 2E).

### 3.4 Subgroup analysis

To assess the predictive performance of our model under different conditions, we conducted subgroup analyses based on sex, age, ethnicity, type of KD, fever days, and illness duration. In each subgroup, the model exhibited good predictive value (Figure 2E). The AUC for illness duration reaching 1 week (0.815) was higher than the AUC for illness duration less than 1 week (0.809); the AUC for KD with fever lasting for 5 days (0.829) was higher than that for fever lasting less than 5 days (0.750); the AUC for IKD (0.897) was significantly higher than the AUC for CKD (0.776); the AUC for Han ethnicity (0.826) was higher than the AUC for ethnic minorities (0.787); and the AUC for males (0.827) was higher than the AUC for females (0.802). Among the various pediatric stages, the AUC for infancy was 0.837, for toddlerhood was 0.808, for preschool age was 0.837, and the lowest AUC for children reaching 7 years was 0.689.

## 4 Discussion

KD is an acute immune vasculitis, and its mechanism remains unclear. As the incidence of CAL increased, early prediction and precise treatment of KD with varying characteristics became increasingly important. Our study conducted a retrospective analysis of the demographic, clinical, and laboratory features of 1,795 KD patients. We constructed a multifactor logistic regression model to predict CAL after variable selection using the LASSO and performed 5-fold cross-validation. We also conducted subgroup validation analyses based on different clinical characteristics. Compared to previous studies, our research utilized a larger KD cohort for model construction while addressing the heterogeneity between KD clinical features and outcomes through subgroup validation.

CAL is a serious complication of KD and a leading cause of acquired heart disease in children. The early pathology of CAL involves acute, self-limiting necrotizing arteritis, typically manifesting within two weeks of KD onset. This condition manifests as neutrophilic inflammation of the vascular endothelium, leading to damage of the intima and other vascular layers (13). Various cytokines and PLT adhere at the injury site, forming saccular aneurysms, which increase the risk of aneurysm rupture or thrombosis. Identifying relevant predictive factors in KD with CAL is crucial. Our study identified WBC, PLT, CRP, ALB, Na, Time to IVIG treatment, and symptoms of limb as significant predictors of CAL. Elevated WBC levels reflected an inflammatory state in the body, indicating exacerbated vascular inflammation and increased CAL risk. Early in KD, PLT counts usually remained within normal ranges or were slightly elevated. However, as the disease progressed, particularly during the second to third week, PLT counts often rose significantly. Previous studies also indicated that elevated PLTs could serve as an independent risk factor for CAL in KD. This may result from vascular inflammation and injury in KD patients, which leads to PLT aggregation at the sites of inflammation and injury (14, 15). When coronary arteries become inflamed, PLTs aggregate extensively, releasing inflammatory mediators such as PLT factors, inflammatory cytokines, and adhesion molecules (16). The massive activation of PLTs may also lead to functional abnormalities, making them more likely to adhere to damaged vascular endothelial cells, thus perpetuating and exacerbating vascular inflammatory responses, further increasing CAL incidence. Notably, PLTs and leukocytes interact, further promoting inflammatory responses and cellular infiltration, exacerbating the degree of vascular inflammation. CRP, the most common inflammatory marker, rises rapidly following inflammation or infection due to the release of cytokines and interleukins that stimulate the liver to synthesize and release CRP (5, 17). Higher CRP levels in KD often indicate more severe vascular inflammation, which increases the likelihood of coronary artery damage. Previous studies found that CAL patients had significantly higher CRP levels than those without CAL (17, 18). IVIG, the first-line treatment for KD, significantly reduced the incidence of CAL; however, a small number of patients still experienced CAL (19–21). Recent studies have also found that hyponatremia is a risk factor for the occurrence of CAL in KD (22). During the acute phase of KD, high levels of cytokines such as IL-6 and TNF-α can directly stimulate the hypothalamus to release antidiuretic hormone, leading to increased renal water reabsorption. This “dilutional hyponatremia” reflects the intensity of the systemic inflammatory response, and severe inflammation is a core trigger for CAL. Additionally, inflammatory factors (such as IL-1β) can directly inhibit the sodium pump function of endothelial cells, resulting in intracellular sodium retention and cellular edema, which disrupts the integrity of the endothelial barrier. This allows lipoproteins and inflammatory cells to more easily infiltrate the vascular wall, promoting the development of coronary artery lesions. First, low albumin weakens antioxidant capacity and increases the release of pro-inflammatory factors (IL-6, TNF-α), which exacerbates endothelial damage and oxidative stress, leading to damage of the coronary artery wall (23). In addition, albumin deficiency promotes a hypercoagulable state (elevated fibrinogen) and microthrombus formation, while impaired NO metabolism triggers vasospasm, collectively aggravating the risk of coronary ischemia and aneurysm. Current research indicates that the timing of IVIG administration correlates closely with the occurrence and duration of CAL. Delayed or insufficient IVIG treatment may prevent timely control of inflammation, thereby increasing CAL risk. Symptoms of limb constitute a novel predictor for CAL in KD patients. Although the precise mechanisms underlying limb involvement in KD remain incompletely understood, we hypothesize that this association likely stems from multiple factors. First, limb changes, such as edema and erythema of the hands and feet, represent a readily apparent localized manifestation of systemic inflammation in KD. The presence of limb symptoms may indicate a more intense systemic inflammatory response, which, in turn, can lead to more severe coronary artery endothelial damage, a critical step in CAL progression. Second, immune complexes formed during KD can deposit in small vessels, including those in the extremities, leading to local vasculitis and tissue injury. These pathological changes may mirror similar processes occurring in coronary microvasculature, thereby promoting CAL development. Furthermore, the release of pro-inflammatory cytokines and vasoactive substances in KD can impair vascular function, resulting in vasoregulatory abnormalities in both the limbs and coronary arteries (10).

In the subgroup analysis, we found that our model had better predictive value for the subgroup characteristics. For the IKD subgroup, IKD is often misdiagnosed or treatment is delayed due to subtle clinical signs, which can easily lead to the occurrence of CAL. Our risk model can help in the early prediction of CAL risk in children suspected of having IKD. It can heighten our vigilance for high-risk populations with CAL, guiding pediatricians to initiate treatment promptly and closely monitor coronary changes in KD patients (24–26). In all age stages of children, the predictive performance of our model was suboptimal for patients over seven years old. This may be due to the relatively mature immune system of children over seven, with more developed immune regulatory mechanisms (such as Treg cell function) that can effectively suppress excessive inflammation (like IL-1β and TNF-α storms) and reduce persistent endothelial damage. Additionally, with increasing age, the proportion of smooth muscle layer and collagen/elastin fibers in the coronary artery wall increases, enhancing vascular mechanical strength and the ability to resist inflammatory damage. In contrast, the vascular walls of infants and toddlers are thinner and have fewer elastic fibers, making them more susceptible to dilation or aneurysm due to inflammation. Moreover, for younger children, symptoms are often not promptly communicated to parents, leading to delays in diagnosis and treatment. This prolonged inflammatory response increases the risk of vascular damage. Additionally, younger patients have underdeveloped vasculature, which weakens their immune resistance and repair capacity, making them more vulnerable to severe vascular damage (27, 28). Therefore, our risk model is particularly effective in identifying high-risk CAL in younger children, especially those under one year of age. It can aid in the early prevention of CAL occurrences and improve long-term outcomes.

In prior studies in China, WBC, PLT, NT-proBNP, DD, ALB, and T cell subsets effectively predicted KD complications like CAL with delayed IVIG treatment (16, 23, 29). Higher WBC and PLT, lower ALB matched our findings, but IVIG was given later in our study, possibly due to delayed recognition of KD in lower-tier hospitals. Our study analyzed Na and other electrolytes (K, Cl, Mg, P, Ca), finding hyponatremia a key predictor for CAL. We identified both hyponatremia and limb symptoms as predictive factors, linking systemic metabolic disturbances to localized vascular inflammation, addressing a gap in CAL prediction related to electrolyte imbalances and clinical symptoms. Though IgA and IgM were not included in the risk model, they were higher in KD patients with CAL, suggesting stronger immune inflammation may lead to CAL. In summary, our study combines indicators of inflammation (CRP and WBC), immune metabolism (ALB and PLT), and treatment (Time to IVIG treatment), laying groundwork for a standardized clinical predictive system.

This retrospective, single-center study is subject to limitations, including potential recall bias. The lack of external validation across diverse regions, ethnicities, and time points also restricts the generalizability of our findings and may impact the predictive performance of the model in broader clinical settings. To address these limitations and enhance the reliability and applicability of future models, we recommend prioritizing large-scale, multi-center, prospective studies incorporating diverse populations.

## 5 Conclusions

In conclusion, our study developed a risk model with good predictive efficacy for CAL in KD patients in China. It also provided robust subgroup validation across age, sex, ethnicity, KD type, fever duration, and disease course. This model serves as a theoretical basis for individualized precision treatment of KD, aiming to improve outcomes and protect the cardiovascular health of KD patients.

## Data Availability

The raw data supporting the conclusions of this article will be made available by the authors, without undue reservation.
